# *Candida glabrata* environmental stress response involves *Saccharomyces cerevisiae* Msn2/4 orthologous transcription factors

**DOI:** 10.1111/j.1365-2958.2008.06301.x

**Published:** 2008-06-16

**Authors:** Andreas Roetzer, Christa Gregori, Ann Marie Jennings, Jessica Quintin, Dominique Ferrandon, Geraldine Butler, Karl Kuchler, Gustav Ammerer, Christoph Schüller

**Affiliations:** 1 University of Vienna, Max F. Perutz Laboratories, Department of BiochemistryA-1030 Vienna, Austria; 2 Medical University Vienna, Max F. Perutz Laboratories, CD Laboratory for Infection BiologyA-1030 Vienna, Austria; 3 School of Biomolecular and Biomedical Science, Conway Institute, University College DublinBelfield, Dublin 4, Ireland; 4 FranceEquipe Fondation Recherche MédicaleUPR 9022 du CNRS, IBMC, 15, rue R. Descartes F67084 Strasbourg Cedex France

## Abstract

We determined the genome-wide environmental stress response (ESR) expression profile of *Candida glabrata*, a human pathogen related to *Saccharomyces cerevisiae*. Despite different habitats, *C. glabrata*, *S. cerevisiae*, *Schizosaccharomyces pombe* and *Candida albicans* have a qualitatively similar ESR. We investigate the function of the *C. glabrata* syntenic orthologues to the ESR transcription factor Msn2. The *C. glabrata* orthologues CgMsn2 and CgMsn4 contain a motif previously referred to as HD1 (homology domain 1) also present in Msn2 orthologues from fungi closely related to *S. cerevisiae*. We show that regions including this motif confer stress-regulated intracellular localization when expressed in *S. cerevisiae*. Site-directed mutagenesis confirms that nuclear export of CgMsn2 in *C. glabrata* requires an intact HD1. Transcript profiles of CgMsn2/4 mutants and CgMsn2 overexpression strains show that they regulate a part of the CgESR. CgMsn2 complements a *S*. *cerevisiae msn2* null mutant and in stressed *C. glabrata* cells, rapidly translocates from the cytosol to the nucleus. CgMsn2 is required for full resistance against severe osmotic stress and rapid and full induction of trehalose synthesis genes (*TPS1*, *TPS2*). Constitutive activation of CgMsn2 is detrimental for *C. glabrata.* These results establish an Msn2-regulated general stress response in *C. glabrata*.

## Introduction

Adaptation of gene expression through regulation of transcription is a key mechanism in fungal response to fluctuating environmental conditions. Environmental stress causes activation of a variety of signalling mechanisms each responding to the particular situation, such as heat shock or osmotic stress, and in parallel evokes a stereotypic general response. In *Saccharomyces cerevisiae*, this response was first described and is referred to as general stress response or environmental stress response (ESR) (Gasch *et al*., 2000; Causton *et al*., 2001). Comparable ESR patterns have been characterized in *Schizosaccharomyces pombe* and to a certain extent in *Candida albicans* (Smith *et al*., 2004; Enjalbert *et al*., 2006; Gasch, 2007). *Candida glabrata* is more closely related to *S. cerevisiae* than *C. albicans* and *S. pombe* (Fitzpatrick *et al*., 2006), and is the second most common fungal pathogen isolated from humans (Kaur *et al*., 2005; Pfaller and Diekema, 2007). Infection rates are relatively low but have been constant during the last decade (Sandven *et al*., 2006). The ESR of *C. glabrata* is currently relatively unexplored.

For *S. cerevisiae*, *C. albicans* and *S. pombe*, one major mechanism for controlling general stress responses are p38-type SAP kinases (stress-activated mitogen-activated protein kinases). The SAPKs, Hog1, Sty1 and CaHog1 are all activated by hyperosmolarity and oxidative stress, and to a varying degree by other stress agents, such as cadmium (Chen *et al*., 2003; Smith *et al*., 2004; Enjalbert *et al*., 2006). The HOG (high osmolarity glycerol) pathway of *C. glabrata* functions in a similar manner to *S. cerevisiae* (Gregori *et al*., 2007).

In *S. cerevisiae*, a second general stress-mediating mechanism based on the transcription factor Msn2 and its paralogue Msn4 exists (Martinez-Pastor *et al*., 1996; Estruch, 2000; Gasch *et al*., 2000; Causton *et al*., 2001; Hohmann, 2002). They are activated by a variety of stress conditions and changing nutrient supply situations, such as the exhaustion of the preferred carbon source glucose (Choo and Klug, 1994; Martinez-Pastor *et al*., 1996; DeRisi *et al*., 1997; Boy-Marcotte *et al*., 1998; Gasch *et al*., 2000; Rep *et al*., 2000; Causton *et al*., 2001; Hasan *et al*., 2002; Cameroni *et al*., 2004; Schüller *et al*., 2004; Teixeira *et al*., 2006). They also have a role in both chronological and replicative ageing (Fabrizio *et al*., 2001; 2004; Powers *et al*., 2006). During high-nutrient supply, Msn2 is inactivated by the PKA (protein kinase A) and TOR (target of rapamycin) pathways (Boy-Marcotte *et al*., 1998; Görner *et al*., 1998; Garreau *et al*., 2000; Görner *et al*., 2002). Activation of Msn2 and Msn4 causes their rapid accumulation in the nucleus and recruitment to chromatin. Msn2 has separate functional domains for nuclear import (nuclear localization signal, NLS), nuclear export (nuclear export signal, NES) and DNA binding. The C_2_H_2_ Zn finger DNA binding domain at the C-terminus recognizes the stress response element (STRE). The NLS is found adjacent to the DNA binding domain; it is phosphorylated and inactivated by PKA when glucose is available and rapidly dephosphorylated and activated by glucose starvation (Görner *et al*., 2002; De Wever *et al*., 2005). Stress signalling requires a region in the N-terminal part of Msn2 which includes its NES and a short stretch of high similarity to Msn4 designated homology domain 1 (HD1) (Görner *et al*., 1998; Görner *et al*., 2002; Durchschlag *et al*., 2004; Boy-Marcotte *et al*., 2006). A variety of stress conditions lead to inhibition of nuclear export of Msn2 by an unknown mechanism. The NES and its surrounding region might therefore represent a crucial determinant for the identification of stress-regulated Msn2 orthologues.

Msn2-like factors do not appear to play a role in regulating the stress response in *C. albicans* and *S. pombe.* The *C. albicans* Msn2 orthologue transcription factor designated CaMsn4 (orf 19.4752) is not involved in the ESR (Nicholls *et al*., 2004). In addition, the Hog1 Map kinase plays a more general role in stress response in *C. albicans* and *S. pombe.* These differences point to distinct strategies for regulating the stress response in different fungi.

Here we investigate ESR transcription patterns of *C. glabrata* and provide evidence that it uses an *S. cerevisiae*-like Msn2-directed stress response. We identify *C. glabrata* Msn2 and Msn4 orthologues based on a motif present in the Msn2/4 NES (HD1) and also in putative Msn2 orthologues of *Ashbya gossypii* and *Kluyveromyces lactis*. Furthermore, we find that *C. glabrata* and *S. cerevisiae* share many common Msn2 target genes. CgMsn2 is required for resistance against severe osmotic stress. In addition, comparison of ESR transcript patterns identifies core similarities and differences between the *S. cerevisiae*, *C. albicans*, *S. pombe* and *C. glabrata* stress responses.

## Results

### The ESR pattern of *C. glabrata* is orthologous to *S. cerevisiae*

The global immediate transcriptional response of *C. glabrata* to a set of environmental conditions was determined via microarray analysis. Conditions chosen were acute carbon starvation by removal of glucose from the medium, mild osmotic stress (0.5 M NaCl), heat stress (42°C) and mild oxidative stress (0.4 mM hydrogen peroxide). The treatment times were 20 min at 30°C to avoid indirect transcriptional responses. Transcript profiles were determined by hybridization to genome-wide *C. glabrata* microarrays. Expression data were filtered and averaged. From the 5063 genes spotted in duplicate, 4166 gave useful data under at least one tested condition. The entire data set was analysed for co-regulated genes by hierarchical clustering (Eisen *et al*., 1998).

**Fig. 1:**
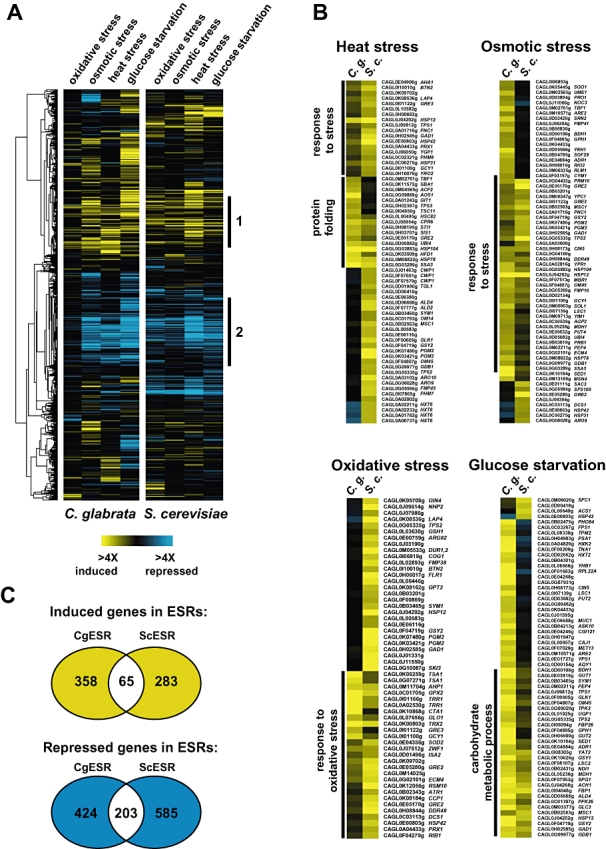
Comparison of genome-wide expression levels in response to environmental changes in *C. glabrata* and *S. cerevisiae*. A. Hierarchical clustering. Transcript profiles were determined by hybridization to genome-wide *C. glabrata* microarrays. The sets represent average inductions of replicate profiles of *C. glabrata* wild-type strain (4166 ORFs) after treatment with 0.4 mM H_2_O_2_, upon glucose starvation, heat shock by incubation at 42°C and hyperosmolarity stress treatment with 0.5 M NaCl (left panel). All treatments were done at 30°C for 20 min. The developed profile was compared with corresponding *S. cerevisiae* expression data (Gasch *et al*., 2000) (right panel). Major clusters are labelled corresponding to induced and to repressed genes (labelled as 1 and 2), in both *C. glabrata* and *S. cerevisiae*. B. Genes involved in four different stress responses were clustered after specific selection (heat stress > 11-fold, osmotic stress > sixfold, oxidative stress > sixfold and glucose starvation > 10-fold). Identified clusters in both *C. glabrata* and *S. cerevisiae* are indicated. Gene names correspond to *C. glabrata* systematic ORF designations and their corresponding *S. cerevisiae* orthologues. C. The overlap between the CgESR and ScESR patterns is depicted as Venn diagram. CgESR was defined as described in Fig. 2A, with genes induced or repressed at least in one tested condition; ScESR data are from Gasch *et al*. (2000).

Similar to the common stress response identified in *S. cerevisiae*, *C. glabrata* has a set of induced and repressed genes common to several stress conditions (Fig. 1A). To compare the *C. glabrata* expression pattern with *S. cerevisiae* data, we used a similarity-based annotation of orthologous genes (Feldmann, 2000) (http://cbi.labri.fr/Genolevures/). Expression data for *S. cerevisiae* exposed to comparable conditions, such as glucose starvation, 0.32 mM H_2_O_2_, heat stress (37°C) and 1 M Sorbitol osmotic stress, were extracted from published ESR data (Gasch *et al*., 2000). *C. glabrata* has a optimal growth temperature of 37°C and was therefore heat-stressed at 42°C. Available evidence suggests that NaCl and Sorbitol are comparable in the concentrations used (Hirasawa *et al*., 2006). Analysis of both ESR data sets highlights clusters corresponding to induced and repressed genes for all environmental conditions (Fig. 1A). This indicates a conserved transcriptional response between *C. glabrata* and *S. cerevisiae*. More detailed comparison of individual stress conditions shows induction of expression of orthologous genes (Fig. 1B). For example, heat stress induces expression of conserved HSP genes in both organisms (*HSP12*, *HSP42*, *HSP78*, *HSP31*, *HSP104*), whereas oxidative stress affects a smaller set of genes in *C. glabrata*. The hydrogen peroxide concentration chosen (0.4 mM) is stressful for *S. cerevisiae* laboratory strains. Furthermore, 0.4 mM H_2_O_2_ *in vitro* corresponds to the *in vivo* oxidative burden in phagocytic cells as judged by the similar transcriptional response of *C. albicans* (Enjalbert *et al*., 2007). However, *C. glabrata* strains are much more resistant to oxidative stress *in vitro* and have a less pronounced response to this concentration. Nevertheless, we find a characteristic pattern of induced genes with functions in oxidative stress response. These include core oxidative stress response genes, such as *TRR1*, *TRX1*, *CTA1*, *SOD1* and *GPX1*.

Glucose starvation and osmotic stress each induce a set of genes orthologous to *S. cerevisiae*. These include *GPH1*, *TPS1*, *TPS2*, *UGP1*, *GSY1* and *GLK1* for glucose starvation and *GRE3*, *PGM2*, *HSP12*, *DDR48* or *SSA3* during osmotic stress. The elevated expression of *TPS1*, *TPS2* and also *TPS3* is perhaps important, as trehalose has a protective role against environmental stresses (Petter and Kwon-Chung, 1996). Taken together, these patterns suggested that the ESR is conserved between *S. cerevisiae* and *C. glabrata*. The overlap between the two ESR patterns is depicted as a Venn diagram of conserved induced and repressed genes (Fig. 1C). The vast majority of repressed genes in both *S. cerevisiae* and *C. glabrata* are involved in ribosome biogenesis. Interestingly and in contrast to *S. cerevisiae*, expression of *C. glabrata* genes involved in sterol biosynthesis (*ERG1*, *ERG2*, *ERG3*, *ERG11*, *ERG13* and *ERG25*) was repressed under all conditions tested. *ERG3* and *ERG11* deletions confer azole resistance (Geber *et al*., 1995).

Some differences between both ESRs are notable. The detailed data are available as supplementary files. *TBF1*, encoding a Telobox-containing general regulatory factor (Bilaud *et al*., 1996), is highly upregulated in *C. glabrata*, whereas it is mainly downregulated in *S. cerevisiae* throughout the conditions compared. *PHO84*, a high-affinity inorganic phosphate transporter and low-affinity manganese transporter (Bun-Ya *et al*., 1991), is downregulated during all stress responses in *S. cerevisiae*, but upregulated in *C. glabrata* during oxidative stress and glucose starvation. The integral membrane protein *VPH2*, required for vacuolar H^+^-ATPase function (Jackson and Stevens, 1997), is highly induced in all tested conditions in *C. glabrata*, whereas it is slightly induced only by oxidative stress in *S. cerevisiae.*

Part of the ESR is also conserved between *C. glabrata*, *S. pombe*, *C. albicans* and *S. cerevisiae.* To compare expression patterns of the four fungi, we used reported orthologous genes between *S. pombe*, *C. albicans* and *S. cerevisiae* (Enjalbert *et al*., 2006). We extended this list by adding the corresponding *C. glabrata* genes with gene similarity data reported by the Genolevures consortium. Data used to generate these figures are available as supplementary files. Comparison of the specific expression profiles revealed a striking overlap between orthologous genes of the individual species for osmotic stress-induced genes (Fig. S2A) and oxidative stress-induced genes (Fig. S2B).

### Orthologues of general stress transcription factors Msn2 and Msn4 in *C. glabrata*

**Fig. 2:**
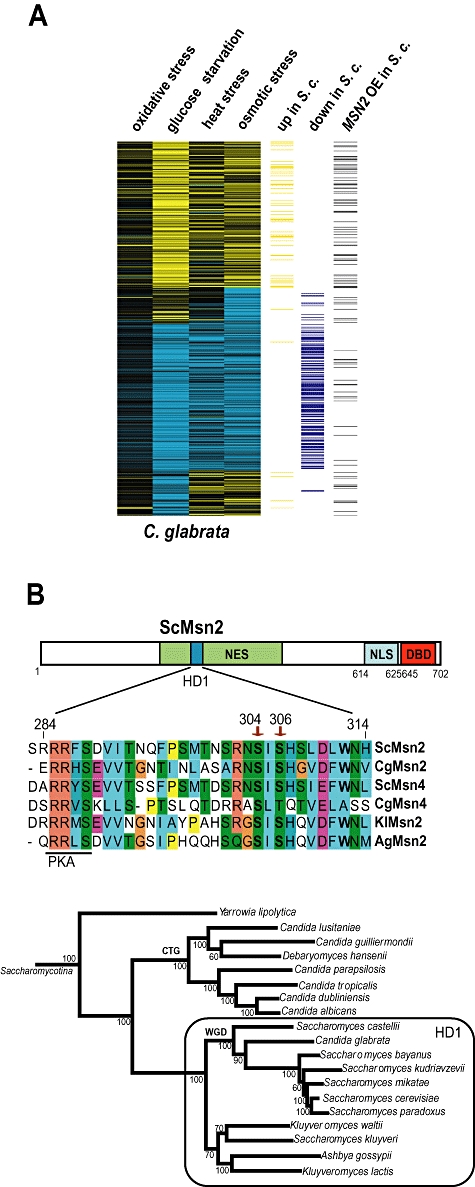
CgESR is similar to ScESR and includes many Msn2-regulated genes. A. The CgESR shown here includes 760 genes selected by being induced or repressed significantly (fourfold) in one of the tested conditions. Columns 5 (up in S.c.) and 6 (down in S.c.) show the corresponding ScESR genes from *S. cerevisiae*. Column 7 (MSN2 OE in S.c.) displays induction of the orthologous *S. cerevisiae* genes by *MSN2* overexpression (Chua *et al*., 2006). B. Alignment of Msn2 orthologous sequences including the HD1. The shared core motif corresponds to positions 284–314 of ScMsn2. The HD1 signature was detected only in close relatives of *S. cerevisiae*, circled in the phylogeny (taken from Fitzpatrick *et al*., 2006). The sequences used in the alignment are: *S. cerevisiae* (YMR037C, ScMsn2; YKL062W, ScMsn4), *A. gossypii* (ABR089C, AgMsn2), *C. glabrata* (CAGL0F05995g, CgMsn2; CAGL0M13189g, CgMsn4) and *K. lactis* (KLLA0F26961g, KlMsn2).

To explore the regulation of the *C. glabrata* ESR (referred hereafter as CgESR), we compared transcription patterns with the *S. cerevisiae* ESR (ScESR). We defined the CgESR by selecting genes from the *C. glabrata* data set which are induced or repressed more than fourfold in at least one condition. This selection resulted in a set of 760 CgESR genes. Of these, 268 genes overlap with the 868 ScESR genes (Gasch *et al*., 2000) (Fig. S3A and S3B). Many of the shared stress-induced genes are induced in *S. cerevisiae* upon overexpression of the general stress transcription factor Msn2 (Chua *et al*., 2006) (Fig. 2A column *MSN2 OE*). Furthermore, Msn2 binding sites (STRE; WAGGGG) are present in many CgESR genes. This suggested that the evolutionary conservation of the ESR is not limited to the set of regulated genes, but also extends to the transcriptional control of those genes.

To identify orthologues of *S. cerevisiae* Msn2 (ScMsn2) in *C. glabrata*, we searched for predicted open reading frames (ORF) comprising C_2_H_2_ Zn-cluster DNA binding domains in available fungal genomes. One further recognizable feature of ScMsn2 is its central region, which confers regulated nuclear export. This is conserved in the paralogue Msn4, and was previously described as HD1 (Görner *et al*., 1998). We detected putative Msn2 (CAGL0F05995g) and Msn4 (CAGL0M13189g) orthologues with HD1 domains at synthenic positions in the *C. glabrata* genome (Fig. 2B). *K. lactis* and *A. gossypii* contain a single orthologue of both genes that also contain an HD1 domain, which we have designated Msn2. The HD1 domain is not present in the single Msn2/4 orthologue in *C. albicans* and related species, nor in similar proteins from *Yarrowia lipolytic*, nor *S. pombe* (Fig. 2B).

**Fig. 3:**
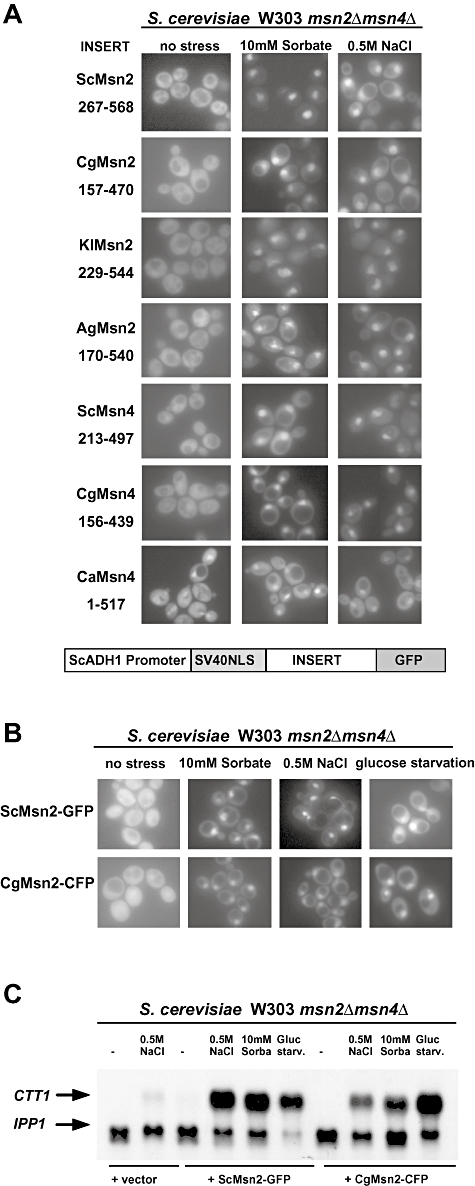
Regulated localization control is functionally conserved in Msn2-like factors. A. Indicated regions of Msn2 and Msn4 orthologues were fused to GFP and a SV40-NLS. GFP fusion plasmids were expressed in *S. cerevisiae* strain W303-1A *msn2Δ msn4Δ*. Localization of GFP fusions was determined by fluorescence microscopy in unstressed cells and 10 min after exposure to weak acid stress (10 mM sorbic acid) and osmotic stress (0.5 M NaCl). B. *S. cerevisiae* W303-1A *msn2Δ msn4Δ* strains containing plasmids expressing either CgMsn2–CFP or ScMsn2–GFP driven by the Sc*ADH1* promoter (pAMG, pACgMC) were grown to exponential phase and exposed to conditions as indicated. Localization was recorded after 10 min by fluorescence microscopy of living cells. C. mRNA levels of the Msn2-regulated gene *CTT1* and the control *IPP1* were visualized on Northern blots after 20 min stress treatment.

**Fig. 4:**
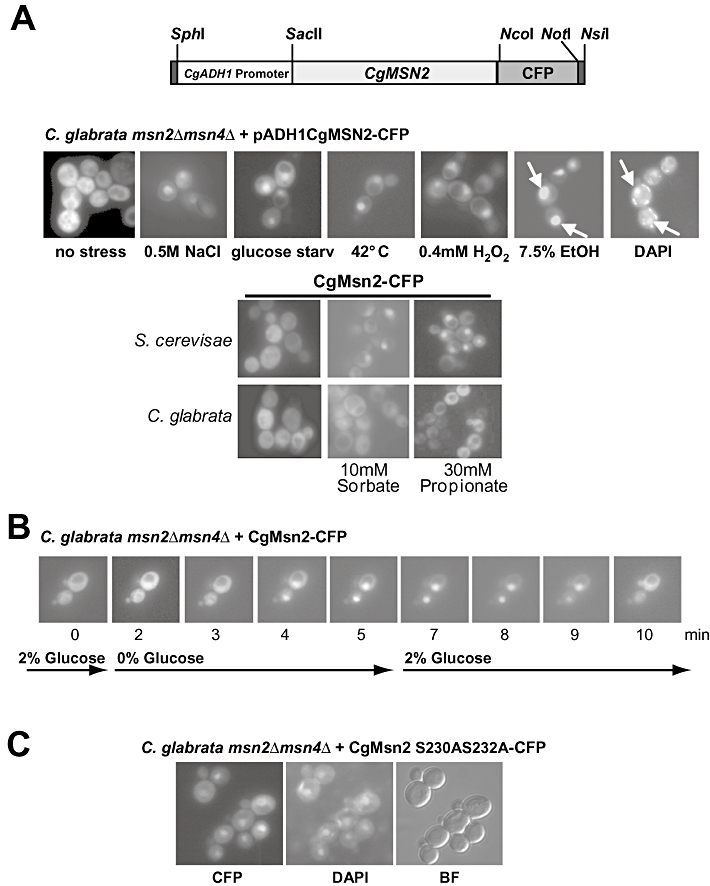

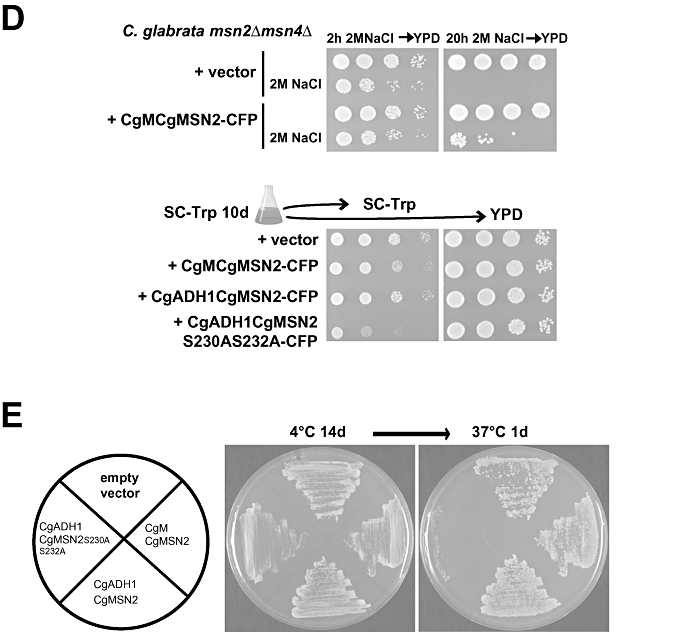
*C. glabrata* CgMsn2 nuclear localization is stress-regulated. A. The CgADH1-*CgMsn2–CFP* construct is illustrated schematically and the positions of used restrictions sites are indicated. Localization of CgMsn2–CFP in the *C. glabrata* strain *Cg msn2Δmsn4Δ* was determined by fluorescence microscopy. CFP fluorescence was recorded in exponentially growing cells approximately 10 min after exposure to the indicated stress conditions. Nuclei were stained by addition of 2 μg ml^−1^ 4,6 diamidino-2-phenylindol (DAPI) dye to the cultures 10 min prior to microscopy. In living cells, nucleic acids, such as the mitochondrial DNA, are also stained by DAPI, resulting in background staining. CgMsn2 localization during weak acid stress. CgMsn2–CFP accumulates in *S. cerevisiae* in the nucleus during weak acid stress (10 mM sorbic acid, 30 mM propionic acid). CgMsn2–CFP does not accumulate in the nucleus in *C. glabrata.* Arrows indicate stained nuclear DNA. B. Nucleocytoplasmic shuttling of CgMsn2–CFP during glucose starvation and re-feeding. Localization of CgMsn2–CFP was visualized by fluorescence microscopy of *C. glabrata* cells fixed to a coverslip with Concanavalin A and localization of the CFP fusion was visualized by fluorescence microscopy. C. Localization of CgMsn2 S230AS232A–CFP in unstressed cells. *Cg msn2*Δ*msn4*Δ cells expressing CgMsn2 S230AS232A–CFP were grown in rich media to early logarithmic phase and localization of the CFP fusion protein was determined by fluorescence microscopy. D. Viability of Cg *msn2*Δ*msn4*Δ mutant cells expressing Msn2 variants. Cultures of *Cg msn2*Δ*msn4*Δ transformed with the empty vector as a control, or with a plasmid expressing *CgMSN2* under the control of the native promoter, were incubated in selective media containing 2 M NaCl for 2 and 20 h. Cell suspensions were spotted in 10-fold dilutions on YPD plates and incubated at 37°C over night. Cultures of *Cg msn2*Δ*msn4*Δ transformed with the empty vector, or with plasmids expressing *CgMSN2* under the control of the native or *CgADH1* promoter, or expressing *CgMSN2 S230AS232A–CFP*, were grown in selective media at 30°C for 10 days. Cells were then spotted in 10-fold dilutions on YPD and SC-Trp plates incubated at 37°C over night and growth recorded. E. High expression of CgMsn2 S230AS232A confers cold sensitivity. Replica-plated patches of *Cg msn2*Δ*msn4*Δ transformed with the above vectors were grown on selective plates overnight at 37°C, plates were kept at 4°C and room temperature as a control for 14 days. Plates were replica-plated to fresh and incubated at 37°C over night and growth recorded. Viability was also tested by colony-forming assay showing a threefold reduced colony-forming ability (0.38 of wild type; SD = 0.12; *P* = 0.05).

Stress-regulated nuclear export of both ScMsn2 and ScMsn4 requires an extended region, including the HD1. To pinpoint the HD1 region of the ScMsn2/4 orthologues as a functional component of the NES, we tested if these regions are sufficient to confer stress-regulated intracellular localization. We expressed parts containing the HD1 but not the NLS and the C-terminal Zn-finger DNA binding domains of the *K. lactis*, *A. gossypii* and *C. glabrata* Msn2 orthologues as GFP fusions in *S. cerevisiae* (Fig. 3A). Sequences were amplified from genomic DNA and fused to an N-terminal nuclear localization signal (SV40NLS) to support constitutive nuclear import. Expression of the fusion genes was driven by the Sc*ADH1* promoter. Cells were grown to early exponential phase, exposed to osmotic stress (0.5 M NaCl) or weak acid stress (10 mM sorbic acid), and the intracellular distribution of the GFP fusion proteins was recorded by fluorescence microscopy. The GFP fusion proteins of the internal regions of Msn2 and Msn4 from *S. cerevisiae* and *C. glabrata*, and Msn2 from *K. lactis* and *A. gossypii*, accumulated rapidly in the nucleus under stress conditions (Fig. 3A). We also analysed the localization of orf19.4752 (CaMsn4), the *C. albicans* orthologue of both ScMsn2 and ScMsn4 (Nicholls *et al*., 2004). CaMsn4 has a similar Zn-finger DNA binding region to the *S. cerevisiae* and *C. glabrata* proteins, but lacks a significant similarity to HD1. The GFP fusion protein comprising the CaMsn4 N-terminal 517 amino acids was constitutively enriched in the nucleus in unstressed cells and stress-induced nuclear accumulation was not detectable. As presented below, point mutations in the conserved residues of the HD1 in CgMsn2 also abolish the NES function (Fig. 4C). These data suggest that regulated nuclear export requires the HD1 domain.

To verify if CgMsn2 functions as a stress-responsive transcription factor, we tested whether CgMsn2 can replace and complement the function of ScMsn2 in *S. cerevisiae*. We expressed full-length *CgMSN2* in a *S. cerevisiae* strain lacking *ScMSN2* and *ScMSN4*. The entire *CgMSN2* reading frame was fused to CFP and its expression driven by the constitutive *ADH1* promoter. In unstressed cells, CgMsn2–CFP was distributed mainly in the cytoplasm, similar to the analogous ScMsn2–GFP protein. Several environmental stress conditions induced rapid nuclear concentration of CgMsn2 in *S. cerevisiae* (Fig. 3B). Next we investigated whether CgMsn2 can complement ScMsn2 and confer stress-regulated gene activation in *S. cerevisiae*. CgMsn2–CFP was expressed in a *S. cerevisiae* strain lacking both *MSN2* and *MSN4* genes (W303 *msn2*Δ *msn4*Δ) exposed to osmotic, weak acid and glucose starvation stress. mRNA levels of a target gene of ScMsn2, the *CTT1* gene coding for catalase T, were analysed by Northern hybridization. As shown in Fig. 3C, CgMsn2 supported the stress induction of *CTT1* transcripts in a very similar manner to ScMsn2.

### CgMsn2 is regulated by stress in *C. glabrata*

These results suggested that CgMsn2 might be the functional orthologue of ScMsn2. To examine the regulation of CgMsn2 in *C. glabrata*, we first analysed its intracellular localization under stress conditions. CgMsn2 was expressed as CgMsn2–CFP fusion driven by the *CgADH1* promoter (Fig. 4A). Live microscopy of CgMsn2–CFP localization revealed a rapid and reversible nuclear accumulation regulated by environmental stress conditions, such as acute glucose starvation, osmotic stress, heat shock, oxidative stress and ethanol stress (Fig. 4A). CgMsn2 activity as a transcriptional activator is not changed by the presence of the C-terminal CFP fusion. In contrast, CgMsn2–CFP failed to accumulate in the nucleus during weak organic acid stress (10 mM sorbic acid, 30 mM propionic acid). Interestingly, CgMsn2–CFP expressed in *S. cerevisiae* accumulates in the nucleus during weak acid stress. This difference in the response of CgMsn2 during weak acid stress could be either a consequence of the higher resistance of *C. glabrata* to weak acids compared with *S. cerevisiae*, which is not the case (Gregori *et al*., 2007), or the absence of a mechanism signalling weak acid stress to CgMsn2 *C. glabrata*.

CgMsn2 accumulates in the nucleus within 4 min following acute carbon source starvation, and subsequent addition of glucose (2%) results in rapid nuclear export (Fig. 4B). The kinetics are very similar to those observed in *S. cerevisiae* (data not shown). To demonstrate that the integrity of the HD1 region is important for NES function, we constructed a mutant derivative by replacing two conserved serine residues S230 and S232 with alanine. The corresponding positions in the ScMsn2 ORF are indicated by arrows in Fig. 2B. As predicted, we find the CgMsn2 S230AS232A-CFP mutant constitutively enriched in the nucleus in unstressed cells, presumably as a result of impaired nuclear export and the basal activity of its nuclear localization signal (Fig. 4C).

We further tested the role of *CgMSN2* for *C. glabrata* survival under extreme osmotic stress. We generated a strain (*Cg msn2*Δ*msn4*Δ) lacking both CgMsn2 and CgMsn4 by homologous gene replacement with the *CgHIS3* and *ScURA3* genes respectively. The correct integrations were confirmed by Southern blot (Fig. S1). The *Cg msn2*Δ*msn4*Δ strain has no obvious growth phenotype under laboratory conditions, similar to *S. cerevisiae msn2Δmsn4*Δ double mutants. Cultures of *Cg msn2Δmsn4*Δ cells transformed with the empty vector as a control, or with a plasmid expressing *CgMsn2–CFP* under the control of the native *CgMSN2* promoter, were grown to early exponential phase and exposed to a severe hyperosmotic stress (2 M NaCl). Viability was assayed after 2 and 20 h incubation by plating of cells in 10-fold serial dilutions. This assay shows that after 2 h of incubation with 2 M NaCl, viability was similar of both strains. However, after 20 h of exposure to 2 M NaCl, cells lacking Msn2 lost viability while the viability of those expressing *CgMSN2* was significantly improved.

Expression of stress genes is tightly regulated and de-regulation has often adverse consequences. We therefore tested if high activity of CgMsn2 compromises long-term viability of *C. glabrata*. *Cg msn2*Δ*msn4*Δ cells transformed with plasmids expressing *CgMSN2–CFP or CgMSN2 S230AS232A–CFP* driven by the *ADH1* promoter were grown for 10 days at 30°C in selective media. Viability was then assayed by spotting 10-fold dilutions. Cells expressing the constitutive nuclear mutant were significantly underrepresented compared with wild type and vector control (about two- to threefold, Fig. 4D, middle panel). The equal cell number on YPD suggests that the *CgMSN2 S230AS232A–CFP* plasmid, but not the other plasmids, was counter-selected in the culture. Cells carrying the mutant plasmid were also cold-sensitive (Fig. 4E).

Taken together, these data demonstrate that nuclear transport of CgMsn2 is highly regulated and requires the integrity of the HD1 region. CgMsn2 is beneficial under extreme stress conditions and its constitutive expression seems to be detrimental.

### The CgMsn2 regulon is related to the ScMsn2 regulon

**Fig. 5:**
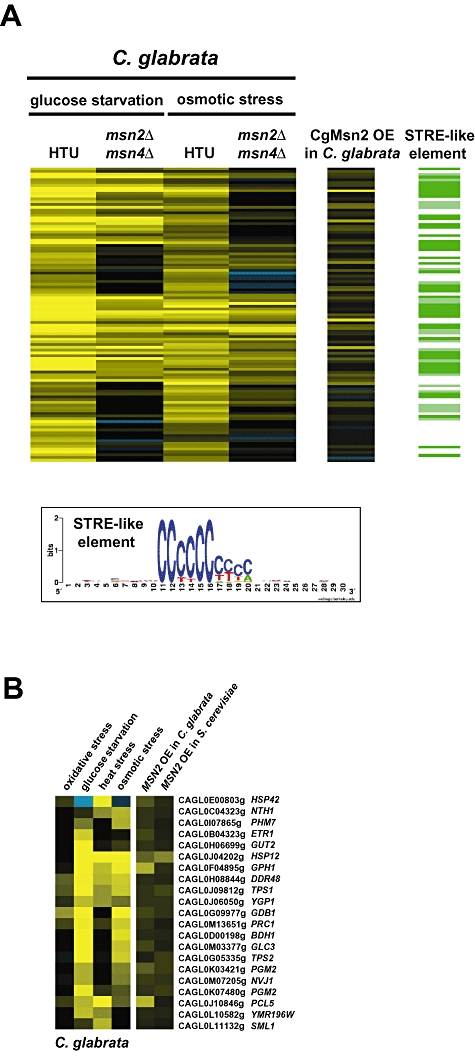
Determination of genes dependent on CgMsn2/CgMsn4. A. Wild type and *Cg msn2Δmsn4Δ* cells were exposed to osmotic stress (0.5 M NaCl) and glucose depletion and transcript profiles determined by hybridization to genome-wide *C. glabrata* microarrays. Hierarchical clustering of genes with a wild type to *Cg msn2*Δ*msn4*Δ ratio > 1.5 is shown. The right panel shows the transcript profile of the *CgADH1* promoter-driven overexpression (OE) of *CgMsn2–CFP* compared with *CgMSN2* native promoter-driven CgMsn2–CFP expression. Logo representation of a STRE-like sequence pattern found by AlignAce among the CgMsn2-regulated genes (indicated on the right). B. *C. glabrata* and *S. cerevisiae* have orthologous Msn2 target genes. A core set of 21 CgESR genes is induced by overexpression of *CgMSN2* in *C. glabrata* and *ScMSN2* in *S. cerevisiae*.

ScMsn2 has about 100–150 target genes depending on the conditions (Gasch *et al*., 2000; Schüller *et al*., 2004). To define the targets of CgMsn2, we compared the expression profiles of *C. glabrata* wild type and *Cg msn2Δmsn4Δ* mutant strains during osmotic stress and acute glucose starvation. We also compared in parallel the mRNA profiles of cells carrying plasmids with the CgMsn2 gene under its own or the *CgADH1* promoter (construct pCgADH1CgMsn2–CFP). Many stress genes influenced by the absence of Msn2 are also induced by the overexpression of *CgMSN2* (Fig. 5A, right panels, supplementary data). Furthermore, we find that *C. glabrata* and *S. cerevisiae* share a set of 21 Msn2 targets that are also part of the 65 induced genes shared between the ScESR and CgESR (Fig. 5B). All the genes found in this core set possess at least one STRE site in their promoters. STRE-like sequences were detected among the CgMsn2-regulated genes by an unbiased heuristic search using AlignAce in the upstream regions of the selected CgMsn2-regulated genes (Hughes *et al*., 2000) and are depicted in the form sequence logo (Fig. 5A, lower panel). The most salient gene functions are connected to stress response (*HSP42*, *HSP12*, *SML1*, *DDR48*), glycogen and trehalose metabolism (*TPS1*, *NTH1*, *TPS2*, *YGP1*) or DNA repair (*SML1*, ribonucleotide reductase) based on GO-terms analysis (*P*-values < 0.005). Detailed data are available as supplementary files. However, genes regulated only in *C. glabrata* by CgMsn2 include the glyoxylate cycle enzyme *MDH3* (cytoplasmic malate dehydrogenase), the glycolytic enzyme *FBP26* (fructose-2,6-bisphosphatase) and *PHM8* involved in phosphate metabolism, suggesting that there is different wiring of some metabolic pathways between the two species. In addition, there are changes in expression of signalling components, such as the casein kinase *YCK1* involved in cell morphogenesis, which are specific to *C. glabrata.* Together, these data show that CgMsn2 has a broad set of target genes and that many of them are conserved between *S. cerevisiae* and *C. glabrata*. A smaller set of CgMsn2 and ScMsn2 target genes are conserved stress genes also in *S. pombe* and *C. albicans* (Fig. S2C). *C. glabrata* is a nicotinamide adenine dinucleotide (NAD+) auxotroph and its growth is dependent on exogenous supply of NAD+ precursors. A main part of the NAD+ metabolism is the Preiss-Handler pathway, where the nicotinamidase Pnc1 ultimately converts precursors to NAD+ (Ma *et al*., 2007). *PNC1* is upregulated in *S. cerevisiae* and *C. glabrata* during oxidative and osmotic stress (Fig. S2C).

### CgMsn2 is required for rapid induction of osmotic stress-induced transcription of trehalose synthesis genes in *C. glabrata*

**Fig. 6:**
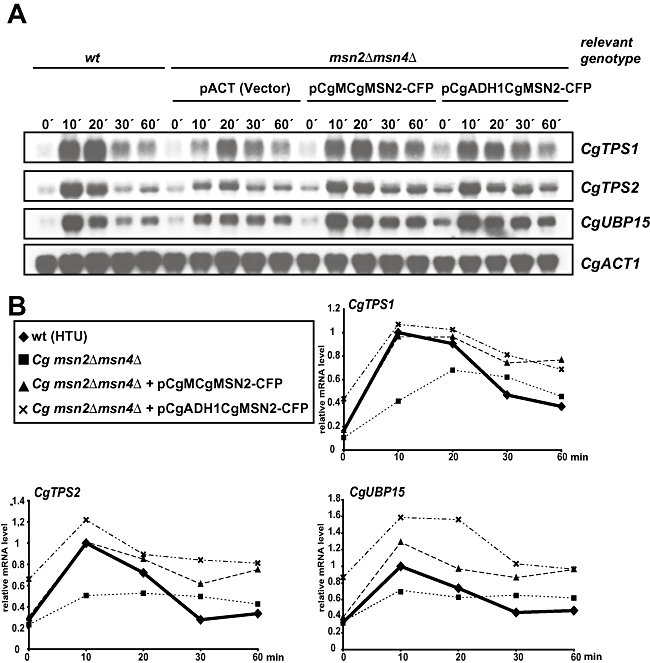
CgMsn2 is required for rapid induction of transcription after osmotic stress. A. Northern blot analysis of *CgTPS1*, *CgTPS2* and *CgUBP15* transcripts during 0.5 M NaCl induced osmotic stress. *C. glabrata* wild type, *Cg msn2*Δ*msn4*Δ and *Cg msn2*Δ*msn4*Δ transformed with pCgMCgMsn2–CFP or pCgADH1CgMsn2–CFP were grown to exponential phase before 0.5 M NaCl was added. Samples for RNA extraction were taken at indicated time points. mRNA levels were visualized by hybridization of radio-labelled probes and autoradiography. *CgACT1* mRNA was used as loading control. B. Quantification of mRNA levels of *CgTPS1*, *CgTPS2* and *CgUBP15* normalized to *CgACT1* and expressed relative to the highest wild-type level.

To confirm the function of CgMsn2 as a stress-regulated transcription factor, we measured the expression of *CgTPS1*, *CgTPS2* and *CgUBP15* over several time points following exposure to osmotic stress. *TPS1* and *TPS2*, encoding trehalose-6-phosphate synthase and phosphatase respectively, are both required for the synthesis and the storage of the carbohydrate trehalose. *UBP15*, coding for an ubiquitin-specific protease, is induced by heat and osmotic stress in *S. cerevisiae*. Northern blot analysis of *CgTPS1*, *CgTPS2* and *CgUBP15* showed rapid induction upon treatment with 0.5 M NaCl (30°C and 37°C gave similar results) in the *C. glabrata* wild type, and also in *C. glabrata msn2*Δ*msn4*Δ mutant cells supplemented with CgMsn2 on a plasmid under its own promoter (pCgMCgMSN2) (Fig. 6A). Cg*ADH1* promoter-driven expression of CgMsn2 in this strain (*C. glabrata msn2*Δ*msn4*Δ, pCgADH1CgMsn2–CFP) enhanced the signals of all genes in untreated and treated conditions. The CFP tag of CgMsn2 did not disrupt its activity (Fig. S4). In the absence of CgMsn2 and CgMsn4, mRNA levels were severely reduced. Stress-induced expression is not completely abolished in the double mutant cells, suggesting the parallel action of other factors reminiscent of *S. cerevisiae* (Reiser *et al*., 1999). These data confirm a direct role for CgMsn2 during osmostress induction. The impact of CgMsn4 remains to be elucidated. We conclude that general stress response mechanisms are at least partially conserved between *C. glabrata* and *S. cerevisiae*.

### CgMsn2 is not required for virulence in a *Drosophila melanogaster* infection model

**Fig. 7:**
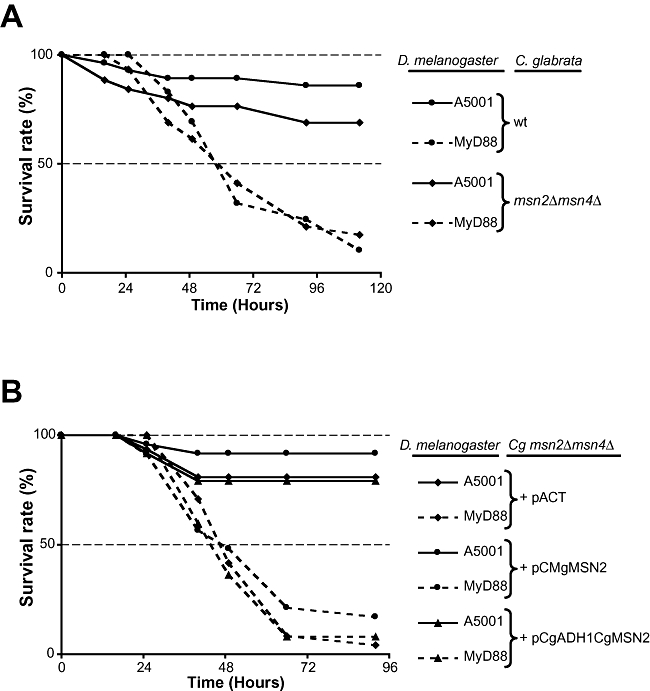
Survival of *D. melanogaster* to wild type or *msn2*Δ*msn4*Δ mutant *C. glabrata* infection. A. Flies were injected with 7500 *C. glabrata* cells. *MyD88* mutant flies succumbed rapidly to a challenge with either wild-type *C. glabrata* or *Cg msn2*Δ*msn4*Δ mutant strain. The genotypes of the infected flies are indicated (A5001: wild-type). B. Immunosuppressed flies succumb rapidly when challenged with *Cg msn2*Δ*msn4*Δ transformed with either the empty pACT plasmid or plasmids expressing CgMsn2 (pCgMCgMSN2 or pCgADH1CgMSN2). Survival was monitored at 29°C. The survival rate is expressed as a percentage. These survival experiments are representative of at least three independent experiments for each panel. The slight difference observed between wild-type flies injected with wild-type or mutant yeasts is not reproducible. Similar results were observed with a lower dose of injected *C. glabrata* (5000).

*Candida glabrata* is a human opportunistic pathogen. To test for a possible role of CgMsn2 in virulence, we tested the *C. glabrata msn2*Δ*msn4*Δ mutant in a *D. melanogaster* model of infection. Wild type flies are resistant to injection of 7500 *C. glabrata* cells (ΔHTU) (Fig. 7). In contrast, *MyD88* mutant flies, in which Toll signalling, and thus the humoral arm of the antifungal response, are blocked, succumb in a few days to the same dose of cells. *C. glabrata* mutants lacking a functional Hog1 pathway have reduced virulence in this infection model, demonstrating its applicability for stress response mutants (not shown). However, we find that there is no difference in the survival of wild-type or immunosuppressed flies when injected either with wild type (ΔHTU) or *Cg msn2*Δ*msn4*Δ mutants. Similarly, we find no difference between the virulence of strains expressing *CgMsn2–CFP* under the control of the endogenous promoter or the strong constitutive *CgADH1* promoter, and the empty control plasmid. These findings are in line with a role of Msn2 during extreme stress situation probably not encountered in the fly hemocoel.

## Discussion

Despite their very different environmental niches and several hundred million years of phylogenetic distance, the transcriptional responses of *S. cerevisiae*, *C. albicans* and *S. pombe* to diverse environmental conditions (ESR) share significant similarities (Enjalbert *et al*., 2006; Gasch, 2007). *C. glabrata* is phylogenetically related to *S. cerevisiae*, but it is adapted to a mammalian host environment. This is reflected by adhesin-mediated adherence to surfaces, the absence of certain biosynthetic pathways (Domergue *et al*., 2005; Kaur *et al*., 2005) and different physiology, for example, optimal growth at 37°C. We report an analysis of part of the CgESR. The CgESR is similar to the ScESR and we identify a conserved role for the general stress transcription factor Msn2.

In many organisms, p38 MAP kinases are central to the control of environmental responses. The *S. pombe* Sty1, *C. albicans* CaHog1 and *S. cerevisiae* Hog1 SAPK mediate the response to a variety of environmental conditions (Toone and Jones, 1998; Wysong *et al*., 1998; Alonso-Monge *et al*., 1999; Hwang *et al*., 2002; Enjalbert *et al*., 2006). *S. pombe* Sty1 is required for most SpESR regulation (Chen *et al*., 2003). *S. cerevisiae* Hog1 is most strongly induced by osmotic stress and activated to lower levels by other stresses, including oxidative stress and weak acid stress (Bilsland *et al*., 2004; Lawrence *et al*., 2004; Sotelo and Rodriguez-Gabriel, 2006). However, many ScESR genes are not exclusively dependent on Hog1, but rely also on the general stress factors Msn2 and Msn4. The *C. albicans* ESR is influenced by CaHog1 and in parallel also by other pathways, but Msn2 does not play any role (Enjalbert *et al*., 2006). This raises the question as to whether *S. cerevisiae* has developed a unique stress response mechanism involving Msn2 and Msn4.

Do functional Msn2 orthologues exist in other ascomycete fungi apart from *S. cerevisiae*? One characteristic feature of Msn2 is its DNA binding domain recognizing the corresponding recognition element STRE. Many fungal genome sequences contain ORFs with high similarity to the Msn2 DNA binding domain. However, these genes usually have very little other sequence conservation to ScMsn2. Zn-finger proteins binding to STRE-like sequences were reported from *S. pombe* and *Trichoderma atroviridae* (Kunitomo *et al*., 2000; Seidl *et al*., 2004), although these factors are not mediating stress responses. The most significant similarity between ScMsn2 orthologues apart from the DNA binding domain (Fig. 2B) is a region designated earlier as Msn2 HD1 (Görner *et al*., 1998). This motif is not present in the Msn2 orthologue from *C. albicans* or its close relatives, including *Candida parapsilosis*, *Candida tropicalis*, *Candida lusitaniae*, *Candida guilliermondii*, *Lodderomyces elongisporus*, *Debaryomyces hansenii* and *Pichia stipitis*.

The intracellular localization of ScMsn2 rapidly changes from the cytoplasm to the nucleus in response to nutrient and stress conditions. (Görner *et al*., 1998; Beck and Hall, 1999; Görner *et al*., 2002). This switch is the result of two independent activities within ScMsn2. The NLS near the C-terminus drives nuclear import. Nuclear export of Msn2 requires the exportin Msn5 which also transports other transcription factors, such as Mig1, Crz1 and Pho4 (Kaffman *et al*., 1998; DeVit, 1999; Chi *et al*., 2001; Boustany and Cyert, 2002). Activation of Msn2 by stress and nutrient starvation requires HD1 which overlaps with the NES function (Görner *et al*., 2002; Boy-Marcotte *et al*., 2006). PKA and TOR inhibit Msn2 activity through this region. Interestingly, all close Msn2 orthologues have an embedded conserved PKA phosphorylation site (Fig. 2B). However, the function of PKA has not been explored in *C. glabrata*.

We have shown that the region including the HD1 motif of Msn2/4 from *K. lactis*, *A. gossypii*, *S. cerevisiae* and *C. glabrata* is sufficient for nuclear export. We provide direct evidence by mutation of two conserved serine residues to alanine in the CgMsn2 HD1, which leads to constitutive nuclear enrichment. Similar changes in the ScMsn2 abolish its NES function (W. Reiter, G. Ammerer and C. Schüller, unpubl. obs.). In contrast, a large part of CaMsn4 (1–517) which is devoid only of its NLS and Zn-finger region at the C-terminus does not support regulated nuclear export.

The NES of ScMsn2 is much larger than the generic exportin 1-driven signal (Wen *et al*., 1995; Kutay and Guttinger, 2005). Importantly, nuclear export of CgMsn2 expressed in *S. cerevisiae* also requires Msn5 and PKA (A. Roetzer and C. Schüller, unpubl. results). These data support our hypothesis that the HD1 is the target site for the exportin Msn5, and that the conserved PKA phosphorylation site within the HD1 site has a regulatory role. Our results suggest that the *Saccharomycotina* can be divided into two groups according to their stress response mechanisms. The HD1 motif and the stress-mediating role of Msn2 orthologues appear after the separation of *C. albicans* from the lineage leading to *S. cerevisiae* (Fig. 2B). The functional conservation of *ScMsn2* and *CgMsn2* highlights the important regulatory motifs of both proteins and will be used as a guide for further structure–function analysis.

*Candida glabrata* as a human commensal and occasional pathogen exists in very different environmental niches compared with *S. cerevisiae*. Our global transcript analysis of *C. glabrata* cells exposed to the generic stress types carbon starvation, heat, osmotic and oxidative stress reveals a transcription pattern related to *C. albicans*, *S. pombe* and *S. cerevisiae*. The most conserved component of the ESRs is dependent on Msn2-like factors in *C. glabrata* and *S. cerevisiae*. Several lines of evidence presented here suggest that CgMsn2 is an orthologue of ScMsn2. It can functionally substitute for ScMsn2 in *S. cerevisiae* for stress-dependent induction of an Msn2 target gene. Intracellular localization of a CgMsn2–CFP fusion protein is stress-regulated in both *S. cerevisiae* and *C. glabrata.* CgMsn2 is required for rapid and full induction of several target genes tested *CgTPS1*, *CgTPS2* and Cg*UBP15* which all contain putative STRE sequences in their promoter region. Conserved Msn2-dependent genes, such as *TPS1*, *NTH1*, *TPS2*, *HSP42*, *HSP12*, *SML1*, *DDR48 and YGP1*, are involved in stress response, glycogen and trehalose metabolism or DNA repair (*SML1*, ribonucleotide reductase) based on GO-terms analysis (*P*-values < 0.005). *C. glabrata* stress genes were also found in a global analysis of the CgPdr1-regulated drug response (Vermitsky *et al*., 2006).

*CgMSN2* and Cg*MSN4* are not required for virulence in a *Drosophila* infection model. This may reflect an absence of extreme stressful conditions for *C. glabrata* in the fly hemocoel. Alternatively, other pathways may allow *C. glabrata* to adapt to the host environment. Finally, the fly model might not reflect all host niches *C. glabrata* is adapted to. Adhesion is essential for *C. glabrata* virulence (Cormack *et al*., 1999). We found no difference of adhesion to a plastic surface (Iraqui *et al*., 2005) in *C. glabrata* Msn2/4 deletion and Msn2 overexpression strains (data not shown). The adhesins encoded by *EPA* genes are regulated differently during environmental stress conditions. Two genes *EPA3*/CAGL0E06688g and CAGL0I00220g are highly induced by osmotic stress and glucose starvation; however, with a minor role for CgMsn2.

Our results highlight the conserved regulation of Msn2 between *S. cerevisiae* and *C. glabrata*, but we also find differences that require further investigation. Stress regulation of many Msn2 target genes is conserved up to *S. pombe*, indicating a selective advantage for such regulation regardless of the particular transcription factors. It will be of particular interest to analyse the role of Msn2 orthologues which we suspect to be stress-regulated factors in the pre-whole-genome duplication clade, such as *Kluyveromyces* species and *A. gossypii*. We suggest that *C. glabrata* Msn2 functions to improve survival under severe stress conditions.

## Experimental procedures

### Yeast strains and plasmids

Yeast strains used in this study are listed in Table 1. Rich medium (YPD) and synthetic medium (SC) supplemented with appropriate auxotrophic components were prepared as described elsewhere (Current Protocols in Molecular Biology; Wiley). Unless otherwise indicated, all strains were grown at 30°C.

**Table 1:** Yeast strains used in this study.

Strain	Genotype	Source
*S. cerevisiae*		
W303-1A	a *ura3 leu2 his3 trp1 ade2 can1*	Nasmyth K.
W303 *msn2Δmsn4Δ*	a *msn2Δ::TRP1 msn4Δ::HIS3*	Görner *et al*. (1998)
*C. glabrata*		
ΔHTU	*his3Δ trp1Δ ura3Δ*	Kitada *et al*. (1995)
*Cg msn2Δ*	*msn2Δ::CgHIS3*	This study
*Cg msn2Δmsn4Δ*	*msn2Δ::CgHIS3 msn4Δ::ScURA3*	This study
*K. lactis*	−	Nasmyth K; Oxford
*C. albicans SC5314*	−	Gillum *et al*. (1984)

Plasmids and oligonucleotides used in this study are listed in Table 2 and Table S1 respectively. *Cg msn2*Δ*msn4*Δ strain was obtained by genomic integration. PCR products of *ScURA3* for *CgMSN4* and *CgHIS3* for *CgMSN4* were amplified from the plasmids pRS316 (Sikorski and Hieter, 1989) and pTW23 (Kamran *et al*., 2004) using fusion PCR (CgMsn4, primer series MSN4; CgMsn2, primer series MSN2) (Noble and Johnson, 2005). Correct genomic integration of all fragments was verified by genomic PCR (primer series Ctrl) followed by Southern analysis using labelled probes generated by amplification with primers MSN2-5′3′/MSN2-5′5′ and MSN4-1/MSN4-3 from genomic DNA.

**Table 2:** Plasmids used in this study.

Plasmids	Relevant Inserts	Source
pAMG	ScADH1-*ScMsn2–GFP* (p*AMG*)	Görner *et al*. (1998)
pSK + CTT1	*ScCTT1* PCR fragment	This study
pACgMC	ScADH1-*CgMsn2–CFP* (*Sal*I/*Nco*I and *Not*I/*Not*I); *ScLEU2* marker.	This study
pCgADH1CgMsn2–CFP	CgADH1-Promoter *CgMsn2–CFP* (SphI/SacII and SacII/NsiI); *CgTRP1* marker.	This study
pCgADH1CgMSN2	CgADH1-Promoter *CgMSN2* (SphI/SacII and SacII/NsiI); *CgTRP1* marker.	This study
pCgMCgMsn2–CFP	CgMSN2-Promoter *CgMsn2–CFP* (SphI/SacII); *CgTRP1* marker.	This study
pCgMCgMSN2	CgMSN2 Promoter *CgMSN2* (SphI/SacII); *CgTRP1* marker.	This study
pCgMSCgMSN2 S230AS232A–CFP	CgMSN2 Promoter-*CgMsn2–CFP* (SphI/SacII); .S230A and S232A, *CgTRP1* marker	This study
pASMG1	ScADH-SV40NLS-ScMsn2–GFP	Görner *et al*. (1998)
pASNScM2G	ScADH1, SV40-NLS, ScMSN2 (267*–*568), GFP; SalI site between SV40 and MSN2 in p*ASMG1*	This study
pASNAgM2G	ScADH1, SV40-NLS, AgMSN2 (170*–*450), GFP	This study
pASNKlM2G	ScADH1, SV40-NLS, KlMSN2 (229*–*544), GFP	This study
pASNCgM2G	ScADH1, SV40-NLS, CgMSN2 (157*–*470), GFP	This study
pASNCgM4G	ScADH1, SV40-NLS, CgMSN4 (156*–*439), GFP	This study
pASNScM4G	ScADH1, SV40-NLS, ScMSN4 (213*–*497), GFP	This study
pASNCaM4G	ScADH1, SV40-Nls, CaMsn3 (1*–*517), GFP	This study
pTW23	pSK^+^ with *CgHIS3*	Haynes K; London
pRS316	CEN6, ARSH4, *ScURA3*	Sikorski and Hieter (1989)
pAMC	ScADH1-ScMSN2-CFP, *ScLEU2*	This study
pAG1334	*MSN2* from *A. gossypii*	Philippsen P; Basel
pACT14	ARS, CEN and *TRP1* marker from *C. glabrata*	Kitada *et al*. (1996)
pGEM-ACT	ARS, CEN and *TRP1* marker from *C. glabrata*	Gregori *et al*. (2007)

Cloned PCR fragments used in this study were confirmed by sequencing. Plasmid pASMG1, which was described in Görner *et al*., (1998), is based on vector YCpLac111 (Gietz and Sugino, 1988) containing the *S. cerevisiae ADH1* promoter followed by the SV40 NLS (PKKKRKV), and a part of *MSN2* coding for amino acid position 267–568 and GFP. To create a unique SalI site after the SV40 NLS, the plasmid was modified by site-directed mutagenesis using a Quick Change Site-directed Mutagenesis Kit (Stratagene) and primers Re-SalI-5/Re-SalI-3 resulting in plasmid pASNScM2G. Sequence fragments from Msn2 orthologues were exchanged with the ScMsn2 sequences by excision with SalI/NcoI. Plasmid pASNKlM2G was created by a SalI/NcoI digest of pASNScM2G and integration of a SalI/NcoI cut fragment obtained by PCR using primers KlM2SalI and KlM2NcoI from genomic *K. lactis* DNA as a template. Plasmid pASNAgM2G was created by using an XhoI/BsmBI fragment of a PCR product generated with primers AgM2XhoI and AgM2NcoI and plasmid pAG1334 as a template. The *C. glabrata* Msn2 and Msn4 orthologues (CAGL0F05995g and CAGL0M13189g) sequences were amplified by PCR from genomic DNA from strain ΔHTU. The CgMsn2 PCR fragment obtained with primers CgM2XhoI and CgM2NcoI was cut with XhoI/NcoI, the CgMsn4 fragment (primers CgM4SalI and CgM4NcoI) was cut with SalI/NcoI and both were inserted into the SalI/NcoI-cut pASNScM2G. pASNScM4G was created by insertion of a SalI/NcoI-digested PCR fragment obtained using primers ScM4SalI and ScM4NcoI and genomic DNA from W303-1A. The CaMsn4 fragment was amplified via PCR (primers CaMsn4-5 and CaMsn4-3) from genomic *C. albicans* DNA and cut with SalI and NcoI. pACgMC was cloned by integration of a SalI/NcoI-cut PCR fragment containing the CgMsn2 ORF into pAMC. pCgACgMsn2–CFP is a derivative of pGEM-ACT (Gregori *et al*., 2007). Nine hundred base pairs of the *CgADH1* promoter were inserted as a SphI/SacII PCR product obtained with primers CgAdhPro-SphI and CgAdhPro-SacII. The coding sequence for CgMsn2–CFP was amplified from pACgMC using primers CgMsn2Cfp-SacII and CgMsn2Cfp-NsiI, and inserted as a SacII/NsiI-cut fragment. The native promoter (860 base pairs) was inserted as a SphI/SacII PCR product obtained with primers SphI-msn2nat and SacII-msn2nat. Exchange of single amino acids in CgMsn2 was done via site-directed mutagenesis using a Quick Change Site-directed Mutagenesis Kit (Stratagene) using the primers 5-SASA and 3-SASA. Probes for Northern and Southern analysis were amplified by PCR from genomic DNA: *IPP1* from *S. cerevisiae*, *CgACT1*, *CgTPS1*, *CgTPS2* and *CgUBP15* from *C. glabrata*. For *ScCTT1*, a KpnI/SacI fragment from the plasmid pKSCTT1 was used.

### Fly strains and survival experiment

Stocks were raised on standard cornmeal-agar medium at 25°C. wA5001 flies were used as wild type throughout the experiments because the MyD88 mutant was generated in this background. Batches of 25–30 wild type and mutant strains were challenged with 7500 cells of wild type or mutant *C. glabrata* using a Nanoject II apparatus (Drummond Scientific). Overnight cultures of *C. glabrata* were collected by centrifugation and washed with PBS 0.01% Tween 20. The solutions were adjusted after counting so that 7500 cells of *C. glabrata* in 13.8 nl were injected into each fly. The quantity of cells injected was checked on a culture plate. After infection, the vials were put in an incubator at 29°C and the surviving flies counted as required. Flies were usually placed into new vials every 2 days. Each infection was carried out using three independent replicates.

### Northern and Southern blot analysis

RNA extraction and analysis followed essentially the protocol as described (Current Protocols In Molecular Biology; Wiley). Cells were grown over night and diluted to an OD_600_ of 0.1 in fresh medium, grown to an OD_600_ of 1 and treated as described. Cells were harvested by centrifugation and frozen immediately. For each RNA extraction, 25 ml of yeast cells was collected. Frozen pellets were re-suspended in RNA isolation buffer (50 mM Tris pH 7.5/5 mM EDTA/5% SDS/130 mM NaCl), 200 μl PCI solution (Roth) and glass beads (2/3 of total volume) were added and total RNA was extracted using FastPrep (2 × 12′′, speed 6, Thermo Savant). RNA samples (20 μg) were separated on a 1.1% agarose gel in FGRB (5×: 0.1 M MOPS/40 mM NaAc pH 7/5 mM EDTA) containing 2.2 M formaldehyde. After transfer to nylon membranes and UV cross-linking, the quality and amount of RNA were determined by staining with Methylene Blue. Hybridization of [^32^P-α]-ATP-labelled probes occurred over night in hybridization buffer (0.5 M sodium phosphate buffer pH 7.2/7% SDS/1 mM EDTA) at 65°C. After washing, the membrane was exposed to an X-ray film. For DNA extraction, yeast cells were grown to an OD_600_ of 6 (10 ml), collected and washed once; cell pellets were re-suspended in Lysis buffer (2% Triton X-100/1% SDS/100 mM NaCl/10 mM Tris pH 8/1 mM EDTA). Genomic DNA was isolated by PCI extraction. Digestion of 10 μg of genomic DNA was done over night with ScaI and BglII (5 U μg^−1^ DNA). The digests were separated and blotted. After cross-linking, the radioactive labelled probes were hybridized over night at 65°C. Bands were detected by X-ray film and PhosphoImager.

### Microscopy

Fluorescence microscopy was performed as described previously (Görner *et al*., 1998). GFP and CFP were visualized in live cells without fixation. Nuclei were stained by addition of 2 μg ml^−1^ 4,6-diamidino-2-phenylindol dye to the cultures 10 min prior to microscopy. All cells were viewed using a Zeiss Axioplan 2 fluorescence microscope. Images were captured with a Spot Pursuit (Sony) CCD camera using MetaVue (Molecular Devices) and Spotbasic software. Time-lapse imaging of life cell was done by adhering the cells to a coverslip with Concanavalin A (Sigma) fixed above a chamber allowing continuous flow of medium driven by a pump. Incoming medium could be switched between two sources containing rich medium without glucose and with 2% glucose. Medium flow rate was about 500 μl min^−1^.

### *Candida glabrata* long-term viability

*Cg msn2*Δ*msn4*Δ transformed with the above plasmids were grown on selective plates, replica-plated to YDP grown over night and then stored for 14 days at 4°C and on room temperature as a control. Plates were then replica-plated and incubated at 37°C over night and growth recorded. Cell patches expressing CgMSN2 S230AS232A–CFP failed to grow, suggesting loss of viability. Viability was also verified by colony-forming assay by plating showing a threefold reduced colony-forming ability (0.38 of wild type; SD = 0.12; *P* = 0.05).

### Microarray analysis

Microarrays were produced (G. Butler lab) by spotting 5908 69- or 70-mer oligonucleotides synthesized at the Pasteur Institute to Corning UltraGAPS slides. Slides were pre-incubated in 0.5% NaBH_4_ (in 75% PBS and 25% EtOH) solution, washed three times with water and dipped in isopropanol. cDNA was synthesized using 15–20 μg of RNA and Superscript III kit (Invitrogen), including either Cy3-dCTP or Cy5-dCTP (Amersham Biosciences). RNA was hydrolysed in 50 mM NaOH at 65°C for 15 min, the solution was neutralized with acetic acid and cDNA was purified using a GFX purification kit (GE Healthcare). Labelled cDNAs were concentrated, pooled and hybridized in 60 μl in DigEasyHyb solution (Roche Diagnostics) with 0.1 mg ml^−1^ salmon sperm DNA (Sigma) at 37°C for 14–16 h. Microarrays were disassembled in 1× SSC, washed two times in 1× SSC, 0.1% SDS at 50°C for 20 min, followed by a 1 min wash in 1× SSC at room temperature. Slides were spun dry for 5 min at 700 r.p.m. Slides were scanned immediately on an Axon4000B scanner (Axon Instruments) and analysed using GenePix Pro4.1 software (Axon Instruments). All standard protocols were provided by the Ontario Cancer Institute (Toronto; http://www.microarrays.ca/). Each experiment was repeated twice.

### Analysis of microarray data

The raw data set of this study is available as supplemental material and has been deposited at array express (http://www.ebi.ac.uk/arrayexpress/; accession number: E-MEXP-1427). Microarrays were analysed with GenePixPro4.1. Values of not found features were excluded from further analysis. Mean ratios were calculated for features with at least four data points and their quality was approximated by their coefficient of variation (CV) values and subsequent exclusion of values with CV smaller than 1. Only genes with at least four data points and a CV > 1 were included in subsequent analysis. Genes assigned as dubious ORFs by the Genolevures consortium analysis were removed from analysis. The filtered median of ratio values were normalized. The normalized values used for further analysis are available as supplementary file *Cg_array_data.xls.* Cluster analysis (Eisen *et al*., 1998) was performed with cluster3 and TreeView (see http://bonsai.ims.u-tokyo.ac.jp/~mdehoon/software/cluster/index.html). Association to GO terms was analysed with the T-Profiler (Boorsma *et al*., 2005) by using the orthologous *S. cerevisiae* genes. Values of genes associated with the most significant terms were visualized by Cluster analysis using complete linkage and correlation as similarity metric. The cluster results were confirmed by K means and SOM clustering. *P*-values of overlapping gene sets were calculated by hypergeometric distribution. Of the 5063 gene features spotted in duplicate on our arrays, 4166 gave useful data under at least one tested condition. Systematic *C. glabrata* IDs were used from the Genolevures resource (5215 ORFs) and linked to systematic names of *S. cerevisiae*. The ESR was defined as the set of genes with values above a fourfold induction and below a fourfold repression after applying different stresses. To estimate the contribution of the transcription factors CgMsn2/CgMsn4, the ratio of the wild type versus double mutant and induced genes was calculated. In addition, data from an all-against-all blastp search of protein sequences from *S. cerevisiae*, *C. albicans* and *S. pombe* (Enjalbert *et al*., 2006) were compared with *C. glabrata* data for osmotic and oxidative stress. Normalized values with canonical gene names are available in plain text format as supplementary data as well as Cluster3 output files corresponding to the figures. Sequence patterns were found by AlignAce (Hughes *et al*., 2000) using the *C. glabrata* genomic DNA. The most recent annotation as of 09.Nov.2007 was used to define ORF and promoter regions. Sequence logo was generated at http://weblogo.berkeley.edu/(Crooks *et al*., 2004).

## Supplementary material

This material is available as part of the online article from: http://www.blackwell-synergy.com/doi/abs/10.1111/

j.1365-2958.2008.06301.x

(This link will take you to the article abstract).
