# Supplementary material for: Candida glabrata environmental stress response involves Saccharomyces cerevisiae Msn2/4 orthologous transcription factors
Source: Mol Microbiol. 2008 Jun 16;69(3):603–20. doi: 10.1111/j.1365-2958.2008.06301.x (PMC2610386; doi:10.1111/j.1365-2958.2008.06301.x)
Supplement: Supplementary file 1 [file mmi0069-0603-SD1.pdf]

Figure S1

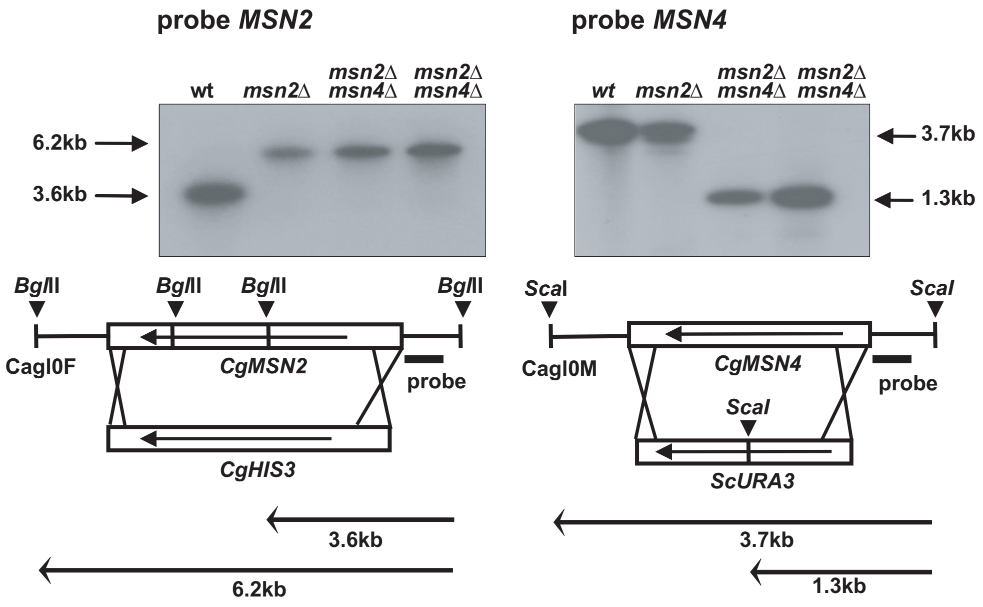
probe MSN2
wt
msn2Δ
msn2Δ msn4Δ
msn2Δ msn4Δ
6.2kb
3.6kb
BglII
BglII
BglII
BglII
Cagl0F
CgMSN2
probe
CgHIS3
3.6kb
6.2kb
probe MSN4
wt
msn2Δ
msn2Δ msn4Δ
msn2Δ msn4Δ
3.7kb
1.3kb
ScaI
ScaI
Cagl0M
CgMSN4
probe
ScaI
ScURA3
3.7kb
1.3kb

Figure S2

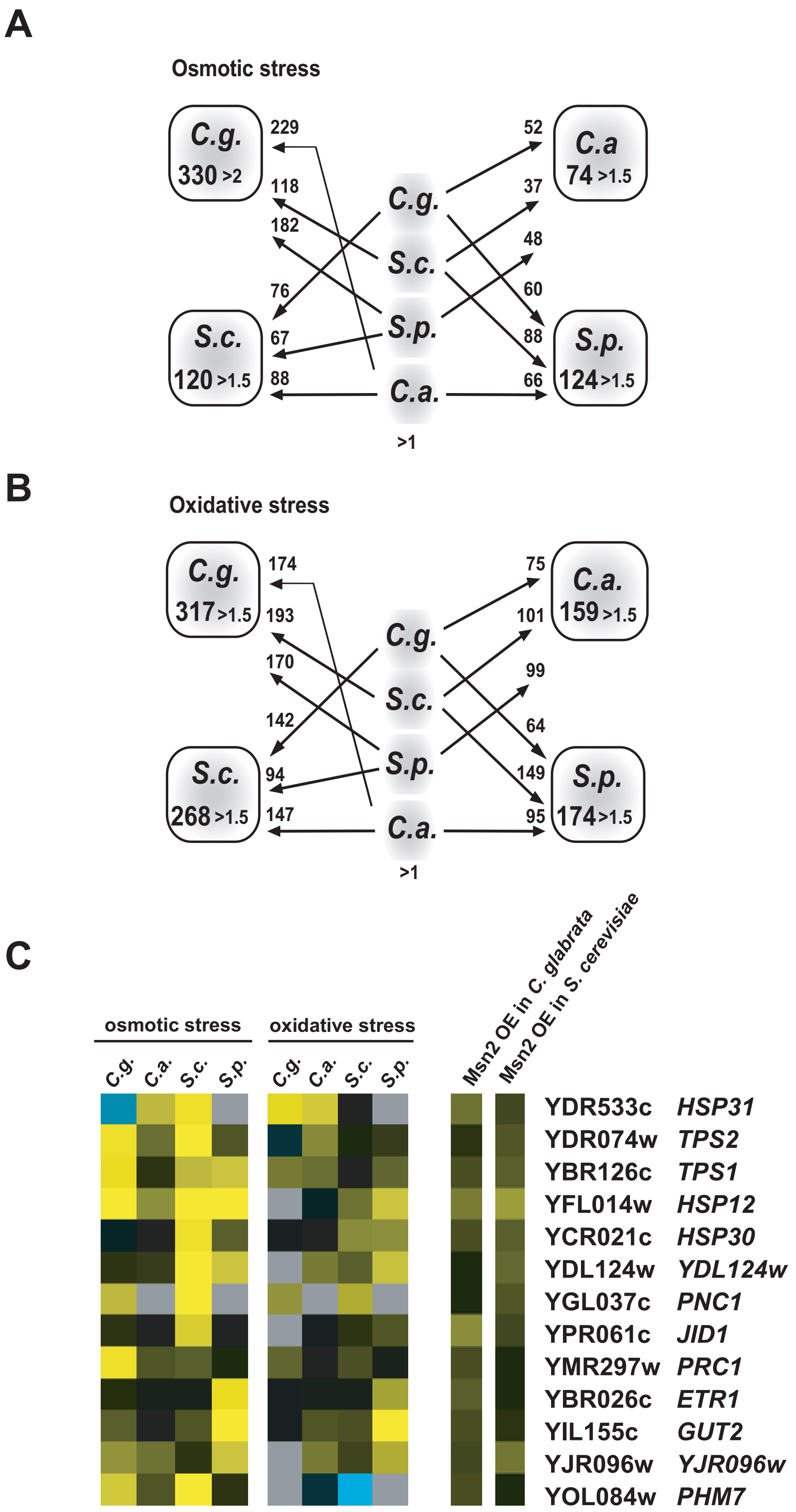
A
Osmotic stress
C.g. 330 >2
C.a 74 >1.5
S.c. 120 >1.5
S.p. 124 >1.5
C.g.
S.c.
S.p.
C.a.
>1
229
118
182
76
67
88
52
37
48
60
88
66
B
Oxidative stress
C.g. 317 >1.5
C.a. 159 >1.5
S.c. 268 >1.5
S.p. 174 >1.5
C.g.
S.c.
S.p.
C.a.
>1
174
193
170
142
94
147
75
101
99
64
149
95
C
osmotic stress
oxidative stress
C.g.
C.a.
S.c.
S.p.
Msn2 OE in C. glabrata
Msn2 OE in S. cerevisiae
YDR533c HSP31
YDR074w TPS2
YBR126c TPS1
YFL014w HSP12
YCR021c HSP30
YDL124w YDL124w
YGL037c PNC1
YPR061c JID1
YMR297w PRC1
YBR026c ETR1
YIL155c GUT2
YJR096w YJR096w
YOL084w PHM7

Figure S3

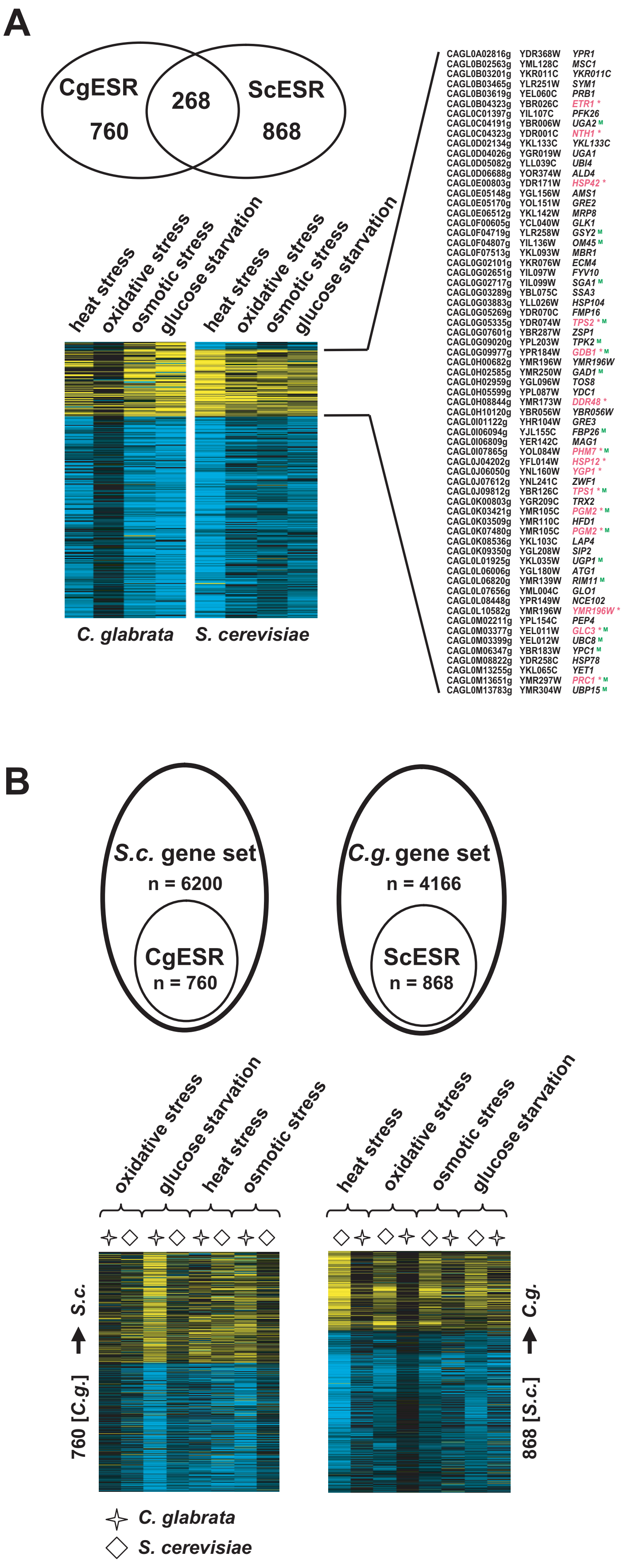
A
CgESR
760
268
ScESR
868
heat stress
oxidative stress
osmotic stress
glucose starvation
heat stress
oxidative stress
osmotic stress
glucose starvation
C. glabrata
S. cerevisiae
CAGL0A02816g YDR368W YPR1
CAGL0B02563g YML128C MSC1
CAGL0B03201g YKR011C YKR011C
CAGL0B03465g YLR251W SYM1
CAGL0B03619g YEL060C PRB1
CAGL0B04323g YBR026C ETR1 *
CAGL0C01397g YIL107C PFK26
CAGL0C04191g YBR006W UGA2 M
CAGL0C04323g YDR001C NTH1 *
CAGL0D02134g YKL133C YKL133C
CAGL0D04026g YGR019W UGA1
CAGL0D05082g YLL039C UBI4
CAGL0D06688g YOR374W ALD4
CAGL0E00803g YDR171W HSP42 *
CAGL0E05148g YGL156W AMS1
CAGL0E05170g YOL151W GRE2
CAGL0E06512g YKL142W MRP8
CAGL0F00605g YCL040W GLK1
CAGL0F04719g YLR258W GSY2 M
CAGL0F04807g YIL136W OM45 M
CAGL0F07513g YKL093W MBR1
CAGL0G02101g YKR076W ECM4
CAGL0G02651g YIL097W FYV10
CAGL0G02717g YIL099W SGA1 M
CAGL0G03289g YBL075C SSA3
CAGL0G03883g YLL026W HSP104
CAGL0G05269g YDR070C FMP16
CAGL0G05335g YDR074W TPS2 * M
CAGL0G07601g YBR287W ZSP1
CAGL0G09020g YPL203W TPK2 M
CAGL0G09977g YPR184W GDB1 * M
CAGL0H00682g YMR196W YMR196W
CAGL0H02585g YMR250W GAD1 M
CAGL0H02959g YGL096W TOS8
CAGL0H05599g YPL087W YDC1
CAGL0H08844g YMR173W DDR48 *
CAGL0H10120g YBR056W YBR056W
CAGL0I01122g YHR104W GRE3
CAGL0I06094g YJL155C FBP26 M
CAGL0I06809g YER142C MAG1
CAGL0I07865g YOL084W PHM7 * M
CAGL0J04202g YFL014W HSP12 *
CAGL0J06050g YNL160W YGP1 *
CAGL0J07612g YNL241C ZWF1
CAGL0J09812g YBR126C TPS1 * M
CAGL0K00803g YGR209C TRX2
CAGL0K03421g YMR105C PGM2 * M
CAGL0K03509g YMR110C HFD1
CAGL0K07480g YMR105C PGM2 * M
CAGL0K08536g YKL103C LAP4
CAGL0K09350g YGL208W SIP2
CAGL0L01925g YKL035W UGP1 M
CAGL0L06006g YGL180W ATG1
CAGL0L06820g YMR139W RIM11 M
CAGL0L07656g YML004C GLO1
CAGL0L08448g YPR149W NCE102
CAGL0L10582g YMR196W YMR196W *
CAGL0M02211g YPL154C PEP4
CAGL0M03377g YEL011W GLC3 * M
CAGL0M03399g YEL012W UBC8 M
CAGL0M06347g YBR183W YPC1 M
CAGL0M08822g YDR258C HSP78
CAGL0M13255g YKL065C YET1
CAGL0M13651g YMR297W PRC1 * M
CAGL0M13783g YMR304W UBP15 M
B
S.c. gene set
n = 6200
CgESR
n = 760
C.g. gene set
n = 4166
ScESR
n = 868
oxidative stress
glucose starvation
heat stress
osmotic stress
heat stress
oxidative stress
osmotic stress
glucose starvation
760 [C.g.] → S.c.
868 [S.c.] → C.g.
C. glabrata
S. cerevisiae

Figure S4

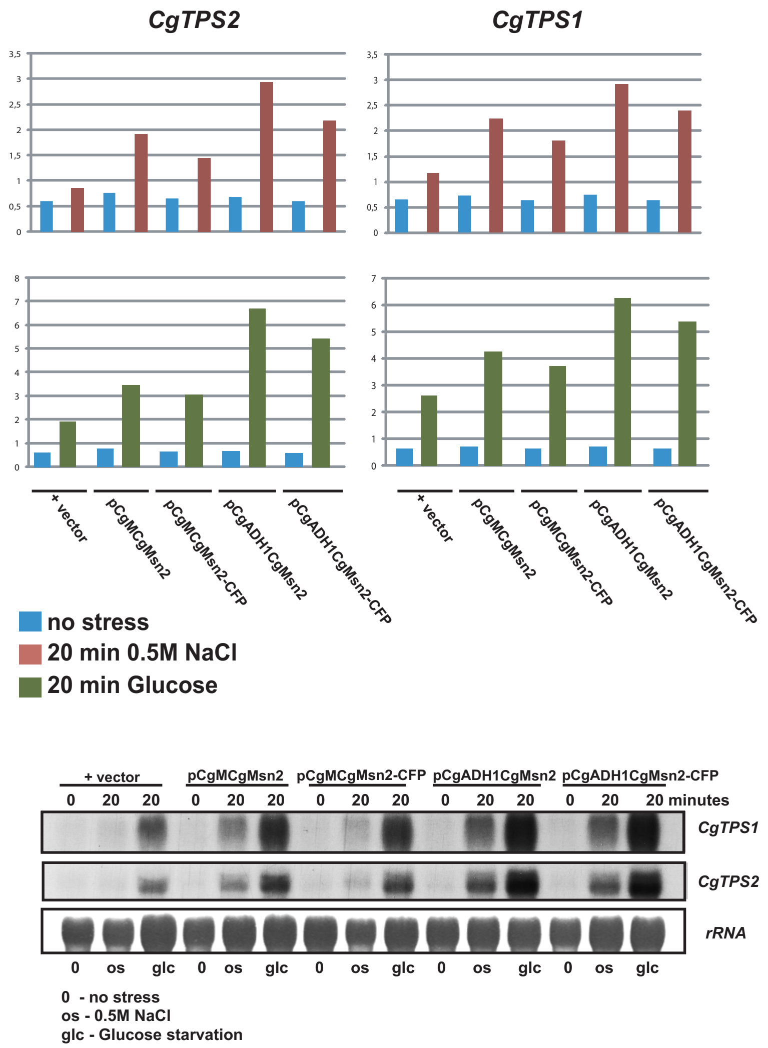
CgTPS2
CgTPS1
+ vector
pCgMCgMsn2
pCgMCgMsn2-CFP
pCgADH1CgMsn2
pCgADH1CgMsn2-CFP
no stress
20 min 0.5M NaCl
20 min Glucose
0 20 20 minutes
CgTPS1
CgTPS2
rRNA
0 os glc
0 - no stress
os - 0.5M NaCl
glc - Glucose starvation

Figure S1. Southern analysis of *CgMSN2* and *CgMSN4* deletion strains. *CgMSN2* was replaced by *CgHIS3* and *CgMSN4* was replaced by *ScURA3.* Genomic DNA from wild type and the *msn2*Δ and *msn2*Δ*msn4*Δ deletion strains was digested with either *Bgl*II or *Sca*I. Chromosomal restriction sites are indicated. DNA fragments were detected with radio-labeled probes as indicated. Replacement of *CgMSN2* with *CgHIS3* strain abolishes two *Bgl*II cleavage sites leading to a 6.2kb fragment instead of the 3.6kb wildtype fragment (left panel). Replacement of *CgMSN4* with *ScURA3* strain introduces a *Sca*I cleavage site changing the 3.7kb wildtype fragment to a 1.3kb fragment (right panel).

Figure S2. ESR gene expression levels of four different yeasts is similar. (A, B) Pairwise overlap of stress induced orthologous genes between *C. glabrata, C. albicans, S. cerevisiae,* and *S. pombe.* Cut-off values are indicated. Expression levels of all four organisms were compared to each other during osmotic **(A)** and oxidative stress **(B).** Specific induction levels were chosen (*C. glabrata* >2-fold after osmotic stress and >1.5-fold upon oxidative stress; *S. cerevisiae* >1.5-fold upon osmotic stress and oxidative stress; *C. albicans* >1,5-fold upon osmotic stress and oxidative stress; *S. pombe* >1,5-fold upon osmotic stress and oxidative stress) and used for a selection among all expressed genes (>1) of the four yeasts during oxidative and osmotic stress, respectively (*C. glabrata*: 945, 1126; *C. albicans*: 934 , 1058; *S. cerevisiae*: 956 , 360; *S. pombe*: 916 , 1065; genes oxidative, osmotic stress). **(C)** Common Msn2 response of orthologous genes. 13 genes are induced significantly (4-fold) by at least one of the tested conditions and by overexpression of ScMsn2 and CgMsn2.

Figure S3. ESR genes are conserved between *C. glabrata* and *S. cerevisiae*. (A) Comparison of genes comprising the CgESR of *C. glabrata* versus the ScESR of *S. cerevisiae*. A set of 268 genes shared between *C. glabrata* and *S. cerevisiae* has a similar stress induction profile for the conditions tested. Color code indicates common Msn2 regulated genes. CgMsn2

regulated genes are highlighted with a green letter (M), whereas genes from core response (see Figure 5B) are in red. **(B)** CgESR from *C. glabrata* (760 genes) is compared to the expression set of *S. cerevisiae* (~ 6200 genes) (left panel), whereas in the right panel the ScESR from *S. cerevisiae* (868 genes) was aligned against the expression set of *C. glabrata* (~ 4200 genes). Data from corresponding stress treatments were chosen: 0.4mM H_2O_2 (*C.g.*) versus 0.32mM H_2O_2 (*S.c.*), 20 minutes of glucose depletion (*C.g.*) versus a corresponding time point of a starvation time course (*S.c.*), temperature shift from 30°C to 42°C (heat shock, *C.g.*) versus shift from 25°C to 37°C (*S.c.*) and 0.5M NaCl (osmotic shock, *C.g.*) versus 1M Sorbitol (*S.c.*). *S. cerevisiae* data were extracted from (Gasch *et al.*, 2000)

Figure S4. CgMsn2 and CgMsn2-CFP display similar rapid induction of transcription after applying stress. (A) mRNA levels of *CgTPS1* and *CgTPS2* were quantified and normalized to large rRNA. **(B)** Northern blot analysis of *CgTPS1* and *CgTPS2* transcripts during 0.5M NaCl induced osmotic stress and glucose starvation. *Cg msn2Δmsn4Δ* transformed with empty vector and *Cg msn2Δmsn4Δ* transformed with pCgMCgMSN2-CFP, pCgMCgMSN2, pCgADH1CgMSN2-CFP and pCgADH1CgMSN2 were grown to exponential phase. Cells were treated with 0.5M NaCl or washed twice and incubated in YP without glucose. Samples for RNA extraction were taken at indicated time points. mRNA levels were visualized by hybridization of radio-labeled probes and autoradiography. Staining of rRNA was used as a loading control.

Table S1 Oligonucleotides used.

Name	Sequence
KlM2SalI	GAG*GTCGAC*AGCTTTGAAGTTACACATTCC
KlM2NcoI	GCCA*CCATGG*AAGTTTGGCTCGTGGAAATTTGAG
AgM2XhoI	AGCTA*CTCGAG*AGTGGCGCAGGCTCGTCG
AgM2NcoI	AAACGTCTC*CCATGG*CGCCCGGAAGGCTTTTTGGATCAAGC
CgM4SalI	GTACT*GTCGAC*TCTAGTCTGGGCAATGAT
CgM4NcoI	CGT*CCATGG*AAGGTGCTAATGACCCTACGC
ScM4SalI	ATCGC*GTCGAC*AGTACAACAGGATTGAACG
ScM4NcoI	ATTT*CCATGG*AAGAGTTCATCGAGATATCATAGC
CgM2XhoI	ATC*CTCGAG*AGCGACCAAAAGGGCAGAAGGG
CgM2NcoI	GCTTT*CCATGG*ATTGGCTATTTTGCCAGGATTCC
CaMsn4-5	GGTACGTCGACATGTCTCAAGAATTCCAACC
CaMsn4-3	GGCTTCCATGGAAGTAGTGATTGGTTTGGTTG
ACT1-5	ATGTGTAAGGCCGGTTTC
ACT1-3	AGGAAGATTGAGCAGCGG
TPS1-5	ATGACACAGATTGAT
TPS1-3	ATTCTGTAGATCTCG
TPS2-5	CGCTACTAAGAAACA
TPS2-3	GTAATCGTGAATCCA
UBP15-5	ATGGGAACAAATGTTGA
UBP15-3	TTATGAAGTTATGCCAC
MSN4-1	AGTTTCTTGCGATACTATAG
MSN4-2	CCGCTGCTAGGCGCGCCGTGAGATTGTACTGAGAGTGCAC
MSN4-3	CACGGCGCGCCTAGCAGCGGAATAAACAAGACTTGCCAAT
MSN4-4	GTCAGCGGCCGCATCCCTGCTTAAAGTCTCGAGCCATTCTT
MSN4-5	GCAGGGATGCGGCCGCTGACCTGTGCGGTATTTCACACCG
MSN4-6	TGTACAAGGAGCAGCATAAA
MSN2-5´M13	ACACAGATCAACAAGAACAGCAGGAAACAGCTATGAC
MSN2-3´M13	TTGTGGAAGTACTTCCCGACCCCAGTCACGACGTTGTA
MSN2-5´3´	GTCATAGCTGTTTCCTGCTGTTCTTGTTGATCTGTGT
MSN2-3´5´	TACAACGTCGTGACTGGGGTCGGGAAGTACTTCCACA
MSN2-5´5´	GTGCGTTGAGAGTTGTCAGCC
MSN2-3´3´	CCAGGTTAGCCTTGTGATGC

5´ExMSN4-Ctrl1	TCACGCCGTGCGAACTC
URA-Ctrl2	TGCTGGCCGCATCTTCT
3´ExMSN4-Ctrl3	TCTCTTTCGGGGGCATC
URA-Ctrl4	TAGTCCTGTTGCTGCCA
5´ÉxMSN2-Ctrl1	CCCGTTTACTCAACAATGAGAC
HIS-Ctrl2	GACACGTAGACTAGCCACAATATC
3´ExMSN2-Ctrl3	TAAGTTCATTGGTCTCTCGG
HIS-Ctrl4	CACTCTACGTAGCAGGCGACCC
CgAdhPro-SphI	AGAT*GCATGC*CAACTTCACAAACACAAACA
CgAdhPro-SacII	CGCA*CCGCGG*TGTTTATGTGTTTTTTGCAG
SphI-msn2nat	AGATGCATGCTATTTTATAGAACAGTAACG
SacII-msn2nat	CGCACCGCGGCTGTTCTTGTTGATCTGTGT
5-SASA	GGCCTCTGCTAGAAATGCTATTGCACACGGTGTCGACTT
3-SASA	AAGTCGACACCGTGTGCAATAGCATTTCTAGCAGAGGCC
CgMsn2Cfp-SacII	CGCA*CCGCGG*ATGACGGTCGACCAGGAGCCT
CgMsn2Cfp-NsiI	AGAGC*ATGCAT*TTATGGGCGGCCGCTCTTGTA
CgMsn2-Cfp1SalI	TTT*GTCGAC*CAGGAGCCTAGCTCGTGG
CgMsn2-Cfp2NcoI	TTAGC*CCATGG*ATTCTTTAGTTGTGGAAGTAC
IPP1 fwd	CCCTTGTACGCTGACAAGG
IPP1 rev	GCTTCACCGGAGAAGGC
Re-SalI-5	GCGGAAGGTGGGGATC*GTCGAC*GTTTCTAGTG
Re-SalI-3	CACTAGAAAC*GTCGAC*GATCCCCACCTTCCGC
